# Proceedings of the Indiana State Medical Society for 1850—1—2—3

**Published:** 1854-07

**Authors:** 


*Proceedings of the Indiana State Medical Society for* 1850—1851—2—3.

THROUGH the Politeness of Dr. C. C. Cornett, we have received a complete set of the Transactions of the Indiana State Medical Society. We find in them, not only evdence of the prosperity and advancement of the Society, but also several articles of value—articles that ought to be read by every Physician in that State. We hope the profession in our sister State will rally still more strongly to the support of their organization, knowing as we do, that every annual gathering together, serves to kindle a new spirit of inquiry, and excite to a nobler aim the generous impulses of the human mind.

